# A statistical strategy for optimizing the production of α-galactosidase by a newly isolated *Aspergillus niger* NRC114 and assessing its efficacy in improving soymilk properties

**DOI:** 10.1186/s43141-022-00315-6

**Published:** 2022-02-25

**Authors:** Ali M. Elshafei, Abdelmageed M. Othman, Maysa A. Elsayed, Gamil E. Ibrahim, Mohamed M. Hassan, Nayra S. Mehanna

**Affiliations:** 1 Microbial Chemistry Department, Biotechnology Research Institute, National Research Centre, 33 El Bohouth St., Dokki, Giza, 12622 Egypt; 2 Chemistry of Flavor and Aroma Department, |Food Industries and Nutrition Research Institute, National Research Centre, 33 El Bohouth St., Dokki, Giza, 12622 Egypt; 3 Dairy Sciences Department, Food Industries and Nutrition Research Institute, National Research Centre, 33 El Bohouth St., Dokki, Giza, 12622 Egypt

**Keywords:** α-Galactosidase, Production optimization, Central composite design, Soymilk treatment

## Abstract

**Background:**

α-Galactosidase is widely distributed in plants, microorganisms, and animals, and it is produced by different fungal sources. Many studies have confirmed the valuable applications of α-galactosidase enzymes for various biotechnological purposes, like the processing of soymilk.

**Results:**

*Aspergillus niger* NRC114 was exploited to produce the extracellular α-galactosidase. One factor per time (OFT) and central composite design (CCD) approaches were applied to determine the optimum parameters and enhance the enzyme production. The CCD model choices of pH 4.73, 1.25% mannose, 0.959% meat extract, and 6-day incubation period have succeeded in obtaining 25.22 U/mL of enzyme compared to the 6.4 U/mL produced using OFT studies. Treatment of soymilk by α-galactosidase caused an increase in total phenols and flavonoids by 27.3% and 19.9%, respectively. Antioxidant measurements revealed a significant increase in the enzyme-treated soymilk. Through HPLC analysis, the appearance of sucrose, fructose, and glucose in the enzyme-treated soymilk was detected due to the degradation of stachyose and raffinose. The main volatile compounds in raw soymilk were acids (45.04%) and aldehydes (34.25%), which showed a remarkable decrease of 7.82% and 20.03% after treatment by α-galactosidase.

**Conclusions:**

To increase α-galactosidase production, the OFT and CCD approaches were used, and CCD was found to be four times more effective than OFT. The produced enzyme proved potent enough to improve the properties of soymilk, avoiding flatulence and undesirable tastes and odors.

**Graphical Abstract:**

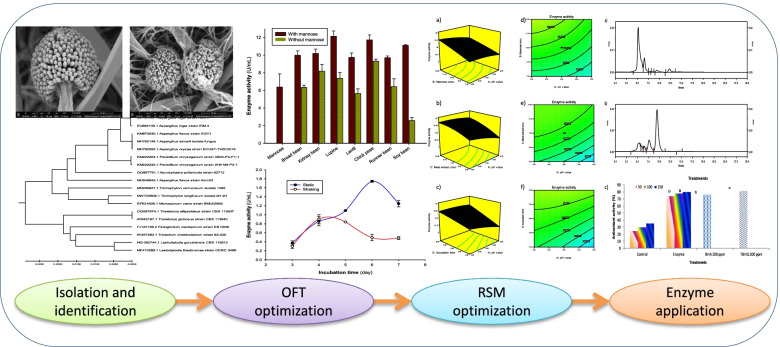

## Introduction

α-Galactosidases (EC 3.2.1.22) are enzymes that degrade the terminal α-linked galactoside residues from melibiose, raffinose, and stachyose, which are the main oligosaccharide sugars in many legumes and their products [1]. The enzyme may have great potential in various applications in the beet sugar industry [2] and soymilk processing [3]. In addition, the enzyme can be exploited in different biomedicine aspects as blood group conversion, treatment of Fabry disease [4], and removal of α-gal-type immunogenic epitopes in xeno-transplantation [5, 6]. α-Galactosidase (α-Gal) is not secreted in humans, and thus the presence of raffinose and stachyose in legumes such as soybeans could hinder digestion and cause flatulence since they are utilized by gas-generating intestinal microorganisms [7]. The use of this enzyme to improve the quality of soy products and other legumes is promising through reducing the anti-nutritional and allergic compounds that exist. Recently, α-Gal was used to treat soybeans components in livestock and poultry meals by minimizing anti-nutritional and inherent factors [8].

α-Galactosidase is widely distributed in plants, microorganisms, and animals. Considering microbial enzymes are more stable than plant and animal enzymes, they serve an important role in the food industry. They may be manufactured in a cost-effective manner with reduced time and space requirements using fermentation processes, and because of their high consistency, process adjustment and optimization can be done very simply [9]. Many of these enzymes have a wide range of uses in a variety of industries. Fungi and their metabolites have substantial industrial uses in high-value-added goods and have the potential to contribute to the creation of nutraceuticals that can enhance health. Furthermore, fungi are well-suited for the creation of natural food additives such as colorants and stabilizers, which pose fewer health concerns than synthetic food additives, as well as bioactive metabolites for pharmaceutical usage such as enzymes, statins, and anticancer agents [10]. In addition, α-galactosidase could be obtained by extraction and purification from germinating guar seeds [11], *Cannabis sativa* L. seeds [12], in bacteria, *Pseudomonas* sp. [13], and *Sulfolobus solfataricus* [14]. Filamentous fungi were found to be good producers of α- Galactosidases due to their ability to grow easily on different agro-wastes. Many studies have confirmed the valuable applications of α-galactosidase fungal enzymes for various biotechnological purposes, like processing of soymilk [15]. Many researchers were interested to produce α-galactosidase from fungal sources such as *Aspergillus oryzae* [16], *Fusarium moniliforme* NCIM 1099 [17], and *Rhizopus oryzae* strain SUK [18].

Optimizing culture conditions is a great way to increase α-galactosidase enzyme output. The one factor at a time technique is the most common method of maximization, although it is time-intensive, difficult, and inefficient. Response surface methodology (RSM) is a collection of mathematical and statistical methodologies that aid in the study of various aspects [19–21]. The relationship between the reaction as well as the independent factors is anonymous in numerous ways; hence, the initial phase in RSM is to quantify the response in light of analyzing the independent parameters. This approach often provides work for a low-order polynomial equation in a predetermined district of the process variables, which will be explored subsequently to select the most appropriate potential values of the input elements for the desired outcome [19, 22]. Numerous technological and biological methodologies have been used successfully in modeling and optimization scenarios to estimate the effective variables and the relationships between various physiological parameters impacting microbial metabolism [23]. RSM has been established and effectively utilized to improve the circumstances and constituents of enzyme fermentation medium by diverse microbial species using statistical methodology [19, 21].

Most of previously reported articles dealt with only one factor per time strategy to optimize the enzyme production from already identified strains, so the existing research aims to optimize the production of α-galactosidase by the local isolated fungal strain *A. niger* NRC114 using RSM statistical approaches. Additionally, the produced enzyme here was applied to improve the nutritional properties of soy milk to make it suitable and palatable for human consumption. Finally, analyses of sugar content, phytochemicals, antioxidant activity, volatile compounds, and sensory evaluation were conducted on the enzymatically treated soymilk.

## Materials and Methods

### Microorganism

A fungal strain was isolated from planted muddy soil (Qalyubia governorate (30.192693, 31.207821)/Egypt). A slant of the pure cultivated isolate was sent to the Macrogen Company (Seoul, South Korea) for sequencing. NS1 and NS8 primers with the sequences “GTAGTCATATGCTTGTCTC” and “TCCGCAGGTTCACCTACGGA” were used to amplify the 18S rRNA. The obtained molecular sequence was registered in the GenBank (NCBI) database, which got an accession number of MW252165. Sequence alignment was done through BLAST on the NCBI portal to find homologous similarities. The registered sequence was compared with other GenBank sequences of high homologous identities, and then sequence alignment and phylogenetic tree were accomplished using MEGA X Software. The strain was identified as *Aspergillus niger* NRC114 and was continuously preserved on Dox^’^s medium to be cultivated on the specific medium for the production of α-galactosidase enzyme.

### Chemicals

*p*-Nitrophenyl α-galactopyranoside (α-p-NPGal) was purchased from Sigma Chemicals Company. Agar was provided by Fluka, Spain. 2, 4, 6-Tri (2-pyridyl)-s-triazine (TPTZ), 1,1^′^-diphenyl-2-picrylhydrazyl (DPPH), butylated hydroxyanisole (BHA), tert-butylated hydroxyl quinone (TBHQ), galactose, glucose, fructose, sucrose, raffinose, and stachyose were purchased from Merck Chemical Company. All the other chemicals used were of analytical grade.

### Media for production of α-galactosidase enzyme

Modified Czapek–Dox’s liquid medium (g/L): NaNO_3_, 2.0; KH_2_PO_4_, 1.0; MgSO_4._7H_2_O, 0.5; KCl, 0.5; glucose or other carbon sources, 20; for 6 days at 28 °C under static and shake incubation conditions. Different leguminous seed powders were added separately by 2% replacing glucose in Czapek–Dox’s liquid medium, namely broad bean, kidney bean, lupine, lentil, chickpeas, runner bean, and soybean.

### Culture conditions and crude enzyme preparations

Erlenmeyer conical flasks (250 mL) each containing 50 mL of sterile medium were inoculated by one agar disc (10 mm) of 7 days old mycelia of *A. niger* NRC114 and incubated at static and shake conditions (150 rpm) using New Brunswick scientific Co. Inc. Edison N. J. USA shaker at 28 °C for 6 days. The grown mycelia were collected, washed with distilled water, and homogenized in citrate phosphate buffer (100 mM; pH 5.0) and cold-washed sand in a cold mortar (4 °C) to obtain the endo-cellular enzyme preparation. The crude homogenate was then centrifuged for 10 min at 5000×*g* and the top layer, which included cell-free extracts, was decanted and utilized as the crude endo-cellular enzyme preparation. On the other hand, broth culture was filtered by Whatman paper no. 1 and used as the extra-cellular enzyme preparation.

### Enzyme assay

α-Galactosidase activity was assayed by the method of Ohtakara et al. [24] using *p*-nitrohenyl α-galactopyranoside (α-*p*-NPGal) as a substrate. The reaction was carried out under assay conditions and was stopped by the addition of 0.2 M sodium carbonate. One unit of α-galactosidase activity is defined as the amount of enzyme capable of releasing one μmole of *p*-nitrophenol in one min. The protein was determined by the method of Bradford [25].

### Optimization of α-galactosidase production using one factor at a time method

To maximize α-galactosidase enzyme synthesis by *A. niger* NRC114, the one factor per time strategy (OFT) was employed to optimize both physiological and culture conditions. This method is based on investigating only one variable per experiment while the remaining parameters remain constant. OFT experimentation was used to examine the effects of cultivation period (3–7 days), pH value of the cultivation medium (pH 3.0-8.0), carbon (mono-, di-, and poly-saccharides) and nitrogen (organic and in-organic) sources and their optimal concentration, and various leguminous seed powders as inducers on *A. niger* NRC114 α-galactosidase production. Following the pre-optimization trials (OFT), four parameters among the most effective factors influencing *A. niger* NRC114 α-galactosidase formation (pH value (*A*), mannose concentration (*B*), meat extract concentration (*C*), and incubation time (*D*)) were chosen to be examined using the CCD technique.

### Experimental design for maximizing enzyme production

To obtain the optimum parameters for producing α-galactosidase, the central composite design (CCD) was used to detect the best values of the major effective factors among numerous self-sufficient variables. In RSM, four stages are followed: trials to shift within the optimal area, the performance of the reaction in the selected region, evaluation of the optimal conditions, and confirmation [26]. CCD was used in the current study, where every changeable factor was measured at four levels (− 2, − 1, + 1, + 2) and midpoint (0), which stands for the central point of each variable series. From our preliminary studies, pH value (*A*), mannose concentration (*B*), meat extract concentration (*C*), and incubation time (*D*) were selected as the most effective variables, and α-galactosidase (Y) was the dependent response parameter. Levels and ranges of the parameters under examination in this study model are presented in Table 1. Experimental runs were considered 2^*k*^ + 2*k* + *x*_0_, where *k* is the quantity of studied variables and *x*_0_ is the midpoint number [27]. Therefore, 30 runs were done in accordance with the CCD given in Table 2.

**Table 1:** The range and levels of the variables

Variable	Symbol	Unit	Range and level of actual and coded values				
			− 2	− 1	0	1	2
pH value	*A*	–	3.5	4.5	5.5	6.5	7.5
Mannose	*B*	(g/L)	5.0	7.5	10.0	12.5	15
Meat extract	*C*	(g/L)	2.5	5.0	7.5	10.0	12.5
Incubation time	*D*	(day)	3	4	5	6	7

**Table 2:** Experimental design and results of the central composite design

Run	pH value	Mannose conc. (g/L)	Meat extract conc. (g/L)	Incubation time (day)	α-Galactosidase activity (U/mL)		
					Actual	Predicted	Residual
**1**	5.5	15	7.5	5	22.528	20.166	2.352
**2**	4.5	12.5	5	6	17.339	17.749	− 0.410
**3**	5.5	10	7.5	5	16.708	16.199	0.509
**4**	4.5	7.5	5	6	10.559	11.684	− 1.124
**5**	5.5	10	7.5	7	22.959	21.118	1.842
**6**	4.5	7.5	10	4	11.692	12.034	− 0.342
**7**	6.5	7.5	5	4	7.023	8.342	− 1.319
**8**	5.5	5	7.5	5	9.046	10.686	− 1.640
**9**	4.5	12.5	10	6	25.199	25.436	− 0.237
**10**	5.5	10	12.5	5	19.059	17.469	1.591
**11**	6.5	12.5	10	4	12.557	12.988	− 0.431
**12**	6.5	7.5	5	6	12.367	10.581	1.786
**13**	5.5	10	7.5	5	15.981	16.199	− 0.218
**14**	5.5	10	7.5	3	8.925	10.055	− 1.130
**15**	6.5	7.5	10	4	10.828	9.574	1.254
**16**	5.5	10	7.5	5	16.327	16.199	0.128
**17**	5.5	10	7.5	5	16.146	16.199	− 0.053
**18**	6.5	7.5	10	6	15.765	16.815	− 1.050
**19**	6.5	12.5	5	4	8.579	7.922	0.658
**20**	3.5	10	7.5	5	16.439	16.696	− 0.257
**21**	6.5	12.5	10	6	23.219	25.552	− 2.333
**22**	4.5	12.5	10	4	15.669	16.612	− 0.942
**23**	4.5	7.5	10	6	15.722	15.536	0.186
**24**	4.5	12.5	5	4	13.422	13.928	− 0.506
**25**	7.5	10	7.5	5	12.938	11.969	0.968
**26**	5.5	10	7.5	5	16.984	16.199	0.785
**27**	6.5	12.5	5	6	14.269	15.483	− 1.214
**28**	5.5	10	2.5	5	7.672	8.551	− 0.879
**29**	5.5	10	7.5	5	15.047	16.199	− 1.151
**30**	4.5	7.5	5	4	16.362	13.185	3.177

### Preparation of soymilk

Soybeans (Majesta variety) provided from Agriculture Research Center (ARC, Egypt) were hydrated (1:3; water: soybean) for 15 h at room temperature and then ground in a crushing machine with heating control (adapted from Frigomat, Milan, Italy) for 20 min at 80 °C with recirculation in a colloidal mill (E. Bachiller B.S.A, Barcelona, Spain). The pulp obtained was separated by filtration (model: CE98, Mejisa e Mectufry, Jijona, Spain).

### Treatment of soymilk by α-galactosidase

The reaction mixture contained 10 mL of enzyme (25.22 U/mL) and 60 mL of soymilk was placed in a 250-mL Erlenmeyer flask. The enzyme reaction was carried out at 50 °C in an incubator shaker (200 rev min^-1^) for an 8-h incubation time. After the incubation period, the reaction mixture was taken out and kept in a boiling water bath for 10 min to restrain the enzyme activity. Afterwards, the produced sugars were assessed.

### Sugar analysis using HPLC

#### Sugars extraction

A treated sample of soymilk by α-galactosidase was deproteinized as described by Mital et al. [28]. Briefly, 0.4 mL of Ba(OH)_2_ (1.8% w/v) was added to 0.2 mL of treated sample and mixed. Afterward, 0.4 mL of ZnSO_4_ (2.0% w/v) was added and the mixture was allowed to stand at room temperature for 10 min, then centrifuged (10000 ×*g* , 10 min, 4 °C) and the supernatant was stored at − 20 °C until analysis.

#### HPLC conditions

The prepared sample was filtered through a 0.45-μm membrane. Analysis of the sugars in the filtrate was performed using HPLC  (Shimadzu Class-VPV 5.03, Kyoto, Japan) equipped with a refractive index RID-10A Shimadzu detector, LC-16ADVP binary pump, DCou-14 A degasser, Shodex PL Hi-PlexPb column (Sc 1011 No. H706081), Guard column Sc-LcShodex, and heater set at 80 °C. Separation and assessment were carried out on an amino-bonded column with a mobile phase of double distilled water at a 1 mL/min flow rate. The identified peaks of the chromatographs were identified by comparing the retention times with the standards samples of galactose, glucose, fructose, sucrose, raffinose, and stachyose.

### Phytochemicals and antioxidant activity measurements

#### Extraction of soymilk samples

Raw soymilk as a control and the treated one with α- galactosidase (5.0 mL) were taken into a 25 mL test tube and extracted using 15 mL ethanol. The solvent layer was separated from the solid residue by centrifuging at 2000×*g* for 10 min. The clear supernatant was transferred to a clean test tube. Then the solid residue was extracted with another 15 mL of ethanol. The separated ethanol layers were combined and dried using a vacuum evaporator at less than 50 °C. The prepared samples extracts were stored at – 20 °C until further analysis.

#### Determination of total phenolic content

Total phenolic contents (TPC) were determined for both raw and enzyme-treated soymilk samples using the Folin–Ciocalteu’s reagent as described by Xiao et al. [29]. Samples were independently analyzed in triplicate and the results were expressed as milligrams of gallic acid equivalents per 100 mL of soymilk (mg GAE/100 mL of soymilk).

#### Determination of total flavanoid

Total flavonoid content was determined by the aluminum chloride method [30] using catechin as a standard. In this regard, the methanol extract (0.1 mL) was added to 0.3 mL of distilled water followed by the addition of 0.03 mL of NaNO_2_ (5% w/v). After 5 min. at 25 °C, AlCl_3_ (0.03 mL, 10%) was added. After a further 5 min., the reaction mixture was treated with 0.2 mL of NaOH (1 mM). Finally, the reaction mixture was diluted to 1 mL with water and the absorbance was measured at 510 nm using a UV–Vis Shimadzu (UV-1601, PC) spectrophotometer. Total flavonoid content was calculated as catechin (mg/100 mL of soymilk).

#### Antioxidant activity analysis

Antioxidant activity was measured using three different methods. The DPPH scavenging activity assay was conducted according to the method of Rani and Pradeep [31]. The FRAP assay was carried out according to the procedure of Guo et al. [32]. The β-carotene bleaching assay was determined according to the method described by Ismail and Tan [33]. As reference compounds, BHA and TBHQ standards at a concentration of 200 ppm were used as benchmarks.

#### Volatile compound analysis

##### Headspace analysis

Agilent Technologies (Palo Alto, CA) headspace (HS) autosampler (7697 A) was used to monitor the static HS quantization of volatiles. The HS-SPME-GC/MS method was used to investigate the volatile compound profile in samples according to the method of Kum et al. [34].

##### GC–MS analysis for soymilk

Analyses were performed on an Agilent 7890 GC coupled to a 5977 MS detector. Manual tuning of the MS with perfluorotributyl amine was used to adjust the relative abundance for *m/z* 69, 219, and 502. The MS was run in the scan mode (*m/z* range from 33 to 400 with a threshold of 100 and a sampling rate of 3 scans/s). Ultrapure helium was passed through moisture and oxygen traps and was used as the carrier gas. The following GC operating conditions were used: a silica capillary column DB-WAX bonded fused capillary column (60-m × 0.25-mm × 0.25-μm film thickness); a flow rate of 1 mL/min at 40 °C; a split ratio of 1:10; the injection port set at 250 °C and the interface line to the MS at 230 °C; and the electron energy and electron multiplier voltage at 70 eV and 1647 V. The temperature of the GC oven was programmed to rise from 40 to 225 °C at a rate of 4 °C/min, with initial and final hold times of 5 and 20 min, respectively.

##### Compound identification

The linear retention index (RI) values for unknowns were determined based on retention time data obtained by analyzing a series of normal alkanes (C_6_–C_22_). Volatile components were positively identified by matching their RI values and mass spectra with those of standards, also run under identical chromatographic conditions in the laboratory [35].

##### Sensory evaluation of the soymilk samples

Soymilk from both the enzyme-treated and control groups was evaluated for their palatability, appearance, flavor, taste, color, and texture. A 9-member panel of judges consisting of members of the Food Industry and Nutrition Research Institute, NRC, rated the samples on a 9-point hedonic scale from 1 to 9, where 1 and 9 represented “dislike extremely” and “like extremely,” respectively [36].

### Statistical analysis

All experimental work was done in triplicate. The results were presented as mean values with standard deviations (SD). Furthermore, MSTAT-C software was used to perform the analysis of variance on the data from the enzymatic treatment of soymilk, and the means were compared using the least significant difference (LSD) test.

## Results

### Isolate identification and localization of α-galactosidase in *A*. *niger* NRC114

**Fig. 1:**
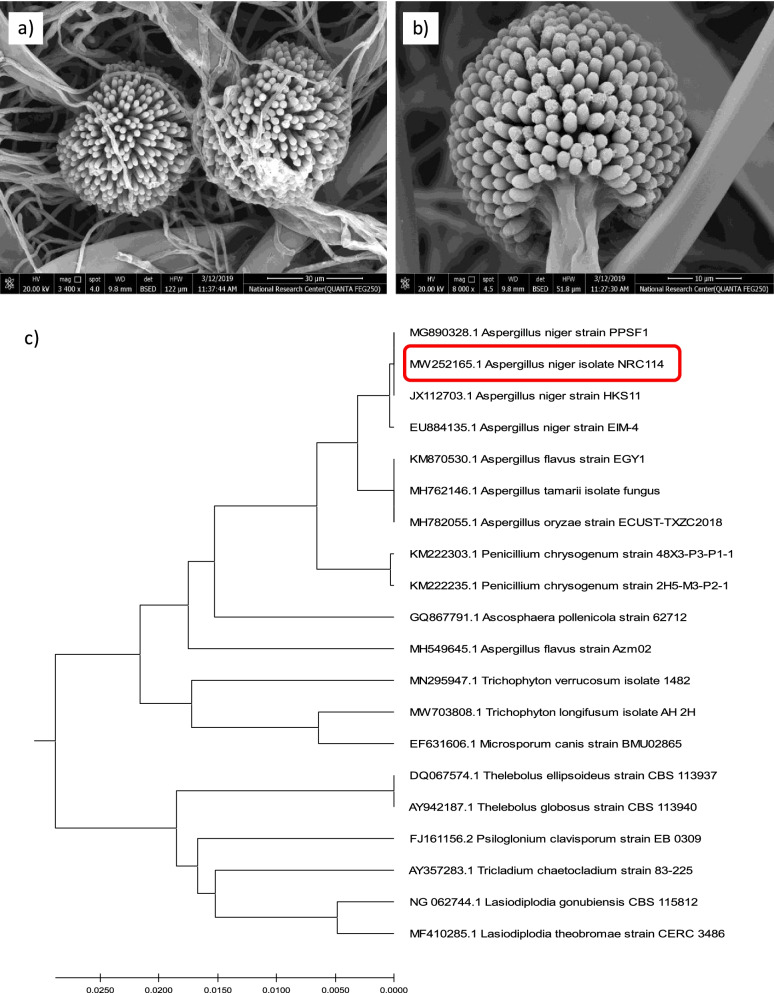
Electron microscope photos (**a**, **b**) and a phylogenetic tree of *A. niger* NRC114 isolate utilizing MEGAX software (**c**)

Developing *Aspergillus niger* conidiophores (fruiting structures) which are characteristic of *Aspergillus* species were recognized by scanning electron micrograph (SEM) (Fig. 1a, b). Images also declare the presence of the conidiophore at the tip of a specialized hypha with a vesicle that contains many nuclei. Additionally, abundant tubular phialides were developed from the vesicle surface. Furthermore, the 18S rRNA sequencing was used to identify the selected isolate that was able to produce α-galactosidase efficiently. Small subunit (18S) rRNA gene sequencing demonstrated the highest homology with other recorded *A. niger* strains in the GenBank (NCBI) database. As demonstrated in Fig. 1c, the phylogenetic tree was built using BLAST data analysis of the isolate’s 18S rRNA region. From these results, the isolate was identified as *A. niger* NRC114 (Accession no. MW252165.1).

The activity of α-galactosidase in *A. niger* NRC114 was estimated in both endo-cellular and extra-cellular preparations to evaluate the extent of the enzyme's localization. Table 3 shows that the enzyme production in the extra-cellular fraction exceeded by about 5.8-fold its presence in the endo-cellular one. In respect of using the enzyme in the treatment of soymilk, it was important to emphasize the absence of aflatoxins in the produced enzyme. Tests for the presence of aflatoxins B1, B2, G1, G2, and ochra-A toxins in the extra-cellular crude preparation clarified that the produced enzyme was free of these toxins (data not shown).

**Table 3:** Levels of the presence of α-galactosidase in *A. niger* NRC114

Type of enzyme	α-Galactosidase activity (U/mL)	Total units
**Endo-cellular**	3.07 ± 0.235	15.35 ± 0.711
**Extra-cellular**	2.53 ± 0.067	88.74 ± 0.182

### Effect of incubation conditions and initial pH values of culuture medium

**Fig. 2:**
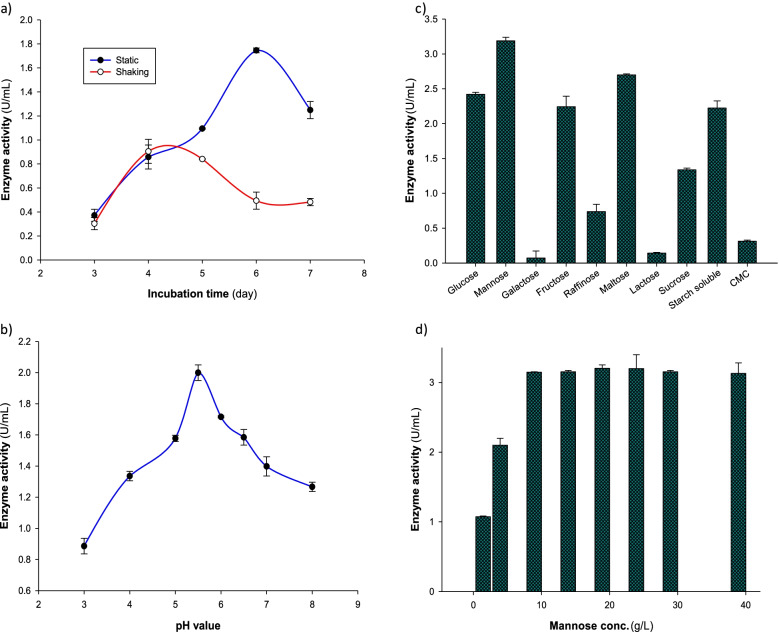
Effect of (**a**) shake and static incubation conditions at different incubation periods, **(b)** different pH values, **(c)** different carbon sources, and (**d)** mannose concentrations on α-galactosidase production by *A. niger* NRC114 isolate

*A. niger* NRC114 was cultivated at 28 °C in static and shaking conditions to detect the most excellent condition for the formation of the enzyme. Figure 2a shows that incubation of the fungus in the static condition provided the formation of the enzyme 4.2-fold greater than in the shaking condition on the sixth day. After that, the production level of the enzyme decreased. The effect of the initial pH value of the culture Dox’s medium on the production of α-galactosidase was examined in the pH range between pH 3.0 and pH 8.0. Figure 2b declares that the enzyme was highly formed at pH 5.5, and then, the enzyme formation was gradually decreased.

### Formation of α-galactosidase as a function of different carbon sources

The effect of nine different carbon sources namely mannose, galactose, fructose, raffinose, maltose, lactose, sucrose, soluble starch, and CMC on the formation α-galactosidase was studied. These compounds were added separately to replace glucose in Dox’s medium at a concentration of 2%. Mannose was superior, followed by maltose, and then glucose to produce the enzyme (Fig. 2c). The figure beside demonstrated that galactose, lactose, and CMC were very poor carbon sources for assisting the formation of the enzyme. As mannose was the superior sugar in producing the enzyme, different concentrations in the range of 2.5–40 g/L of this sugar were tested. Figure 2d demonstrates that the enzyme productivity increased by the increase of mannose until it reached 1.0%, after which the produced enzyme level was more or less the same.

### Formation of α-galactosidase as a function of different nitrogen sources

**Fig. 3:**
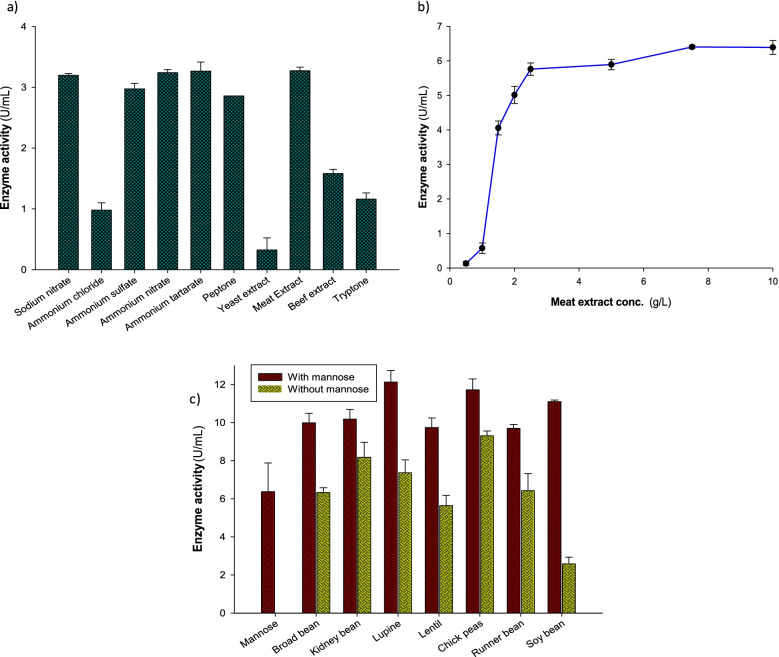
Effect of (**a**) different nitrogen sources, **(b)** meat extract concentrations, and (**c**) addition of leguminous seed powders in the presence and absence of a fixed concentration of mannose (1%) on the production of *A. niger* NRC114 α-galactosidase

The Dox’s medium contained mannose as a carbon source was supplemented by some inorganic nitrogen sources containing the equivalent weight of nitrogen (0.033% as nitrogen base), and also five organic nitrogen sources were added separately. The results presented in Fig. 3a clearly shows that the type of various nitrogen sources, whether inorganic or organic (sodium nitrate, ammonium nitrate, ammonium tartarate, or meat extract) caused more or less the similar effect (3.19–3.26 U/mL) on the production of α-galactosidase, while yeast extract caused the lower productivity of the enzyme (0.32 U/mL). The effect of different concentrations of meat extract on the enzyme production level is demonstrated in Fig. 3b. The figure clarifies that α-galactosidase formation increased by increasing meat extract concentration in the culture medium and reached its higher level at the concentration of 7.5 g/L, while with the addition of more quantity of meat extract, the formation of the enzyme was at the same level (6.4 U/mL).

### Effect of addition of leguminous seed powders

Seven different leguminous seed powders were added separately at a final concentration of 2% to the basal medium in the absence and presence of 1% mannose. The results in Fig. 3c show that the addition of chickpea only caused the highest formation of α-galactosidase, followed by kidney bean powder and lupine powder. Otherwise, incorporation of mannose at 1.0% to leguminous powders medium caused the enhancement of α-galactosidase production. Figure 3c also clarifies the integration effect of the addition of mannose to the medium containing leguminous powders on increasing the production of the enzyme, which was represented at the highest level with lupine powder.

### Maximization of α-galactosidase production by using RSM-CCD

Trial studies were performed to produce the highest production level of α-galactosidase by using a single factor per time (Figs. 2 and 3). The previous results revealed that incubation time on the sixth day, pH value of 5.5, mannose at a concentration of 1.0%, and meat extract at a concentration of 0.75% could influence the increase in enzyme production by 1.8, 2.02, 3.18, and 6.4 U/mL, respectively. So, the response surface methodology was designed using the values of effective parameters (as obtained from one factor per time study) that influence the secretion of *A. niger* NRC114 α-galactosidase.

ANOVA analysis of the RSM-CCD design (Table 4) revealed that the model *F*-value of 13.12 implies that the model is significant, where there is only a 0.01% chance that a “model *F*-value” this large could occur due to noise. Additionally, values of “prob > F” that are less than 0.05 indicate model terms are significant, and in the current case, pH value (*A*), mannose concentration (*B*), meat extract concentration (*C*), incubation time (*D*), *BC*, *BD*, *CD*, and *C*^2^ are significant model terms. On the other hand, values greater than 0.1 indicate that the model terms are not significant. Analysis results have an *R*-squared value of 0.9244 and an “adj *R*-squared” of 0.8540. “Adeq precision” measures the signal-to-noise ratio, where a ratio greater than 4.0 is desirable. Here, the ratio of 14.014 indicates an adequate signal and this model can be used to navigate the design space. The final equation in terms of coded factors is:

$$\mathrm{Enzyme}\ \mathrm{activity}=16.20-1.18\ast A+2.37\ast B+2.23\ast C+2.77\ast D-0.29\ast A\ast B+0.60\ast A\ast C+0.93\ast A\ast D+0.96\ast B\ast C+1.33\ast B\ast D+1.25\ast C\ast D-0.47\ast {A}^2-0.19\ast {B}^2-0.80\ast {C}^2-0.15\ast {D}^2$$

**Table 4:** Analysis of variance table for RSM-CCD

Source	Sum of squares	df	Mean square	***F***-value	***p***-value (prob > *F*)
Model	581.1992	14	41.51423	13.11533	< 0.0001
*A*—pH value	33.51662	1	33.51662	10.5887	0.0053
*B*—Mannose conc.	134.8032	1	134.8032	42.58754	< 0.0001
*C*—Meat extract conc.	119.2925	1	119.2925	37.68734	< 0.0001
*D*—Incubation time	183.5619	1	183.5619	57.99156	< 0.0001
*AB*	1.352597	1	1.352597	0.427317	0.5232
*AC*	5.675014	1	5.675014	1.792872	0.2005
*AD*	13.98608	1	13.98608	4.418535	0.0529
*BC*	14.70656	1	14.70656	4.646151	0.0478
*BD*	28.32565	1	28.32565	8.948746	0.0091
*CD*	25.02249	1	25.02249	7.905199	0.0131
*A*^2^	5.970958	1	5.970958	1.886368	0.1898
*B*^2^	1.022902	1	1.022902	0.323159	0.5781
*C*^2^	17.43682	1	17.43682	5.508706	0.0331
*D*^2^	0.643106	1	0.643106	0.203172	0.6586
Residual	47.47981	15	3.16532		
Lack of fit	45.21154	10	4.521154	9.966101	0.0102
Pure error	2.268266	5	0.453653		
Cor total	628.6791	29			

Std. dev., 1.78; *R*-squared, 0.924; adj *R*-squared, 0.854; C.V.%, 11.932; adeq precision, 14.014

**Fig. 4:**
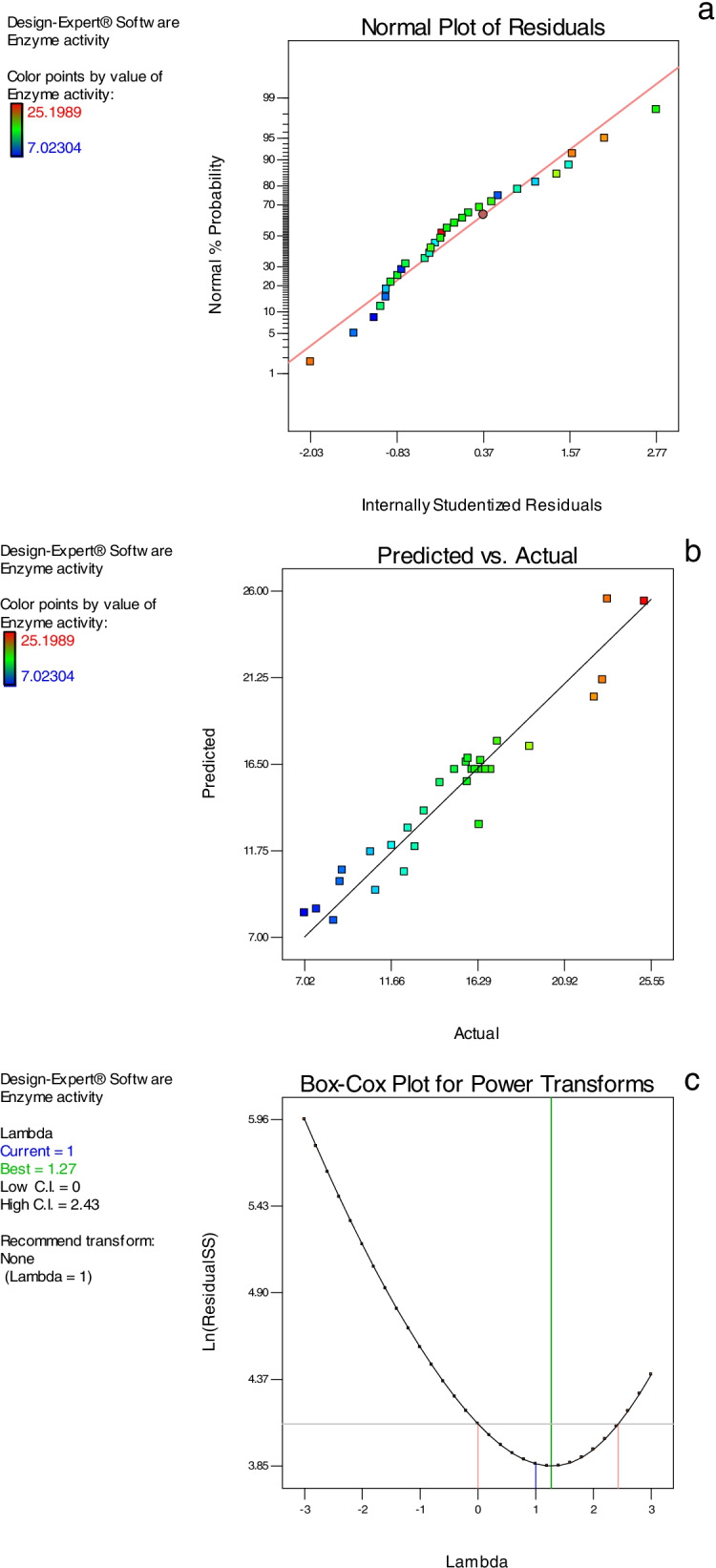
**(a)** The normal probability plot of residuals of the predictable *A. niger* NRC114 α-galactosidase secretion, (**b)** predicted and actual values plot, and **(c)** Box–Cox plot for power transforms

The normal probability plot of the residuals is considered a noteworthy way to explain the normality of data (Fig. 4a). The normality plot demonstrated that the relationship points are near to the diagonal line and the residuals are disseminated normally. Additionally, predicted and actual values plots are presented in Fig. 4b to compare both predicted and actual values of the enzyme activity. The Box–Cox transformation (Fig. 4c) is a way to develop uniformity of dispersion by promoting it to the power and adjusting it for statistical analysis.

**Fig. 5:**
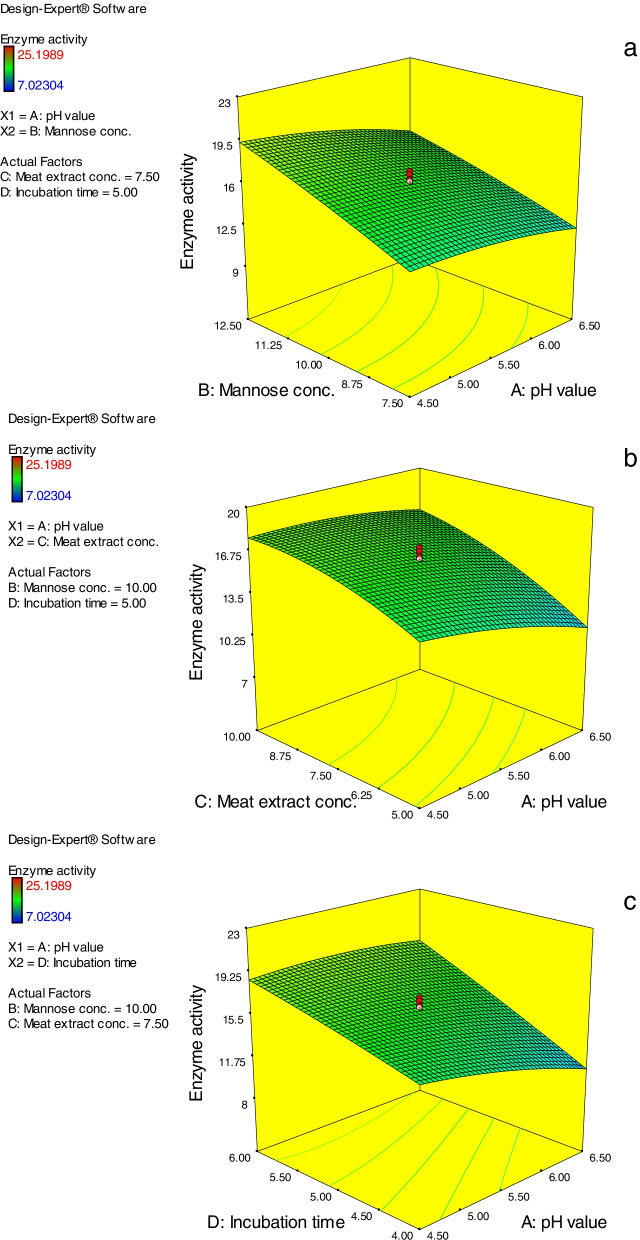
**(a**–**c)** Three-dimensional surface models of *A. niger* NRC114 α-galactosidase formation. For each figure, the absent two parameters were fixed at 0 levels (i.e. pH value, 5.5; mannose conc., 10 g/L; meat extract, 7.5 g/L; and incubation time, 5 days) while the effect of the other two variables was measured

**Fig. 6:**
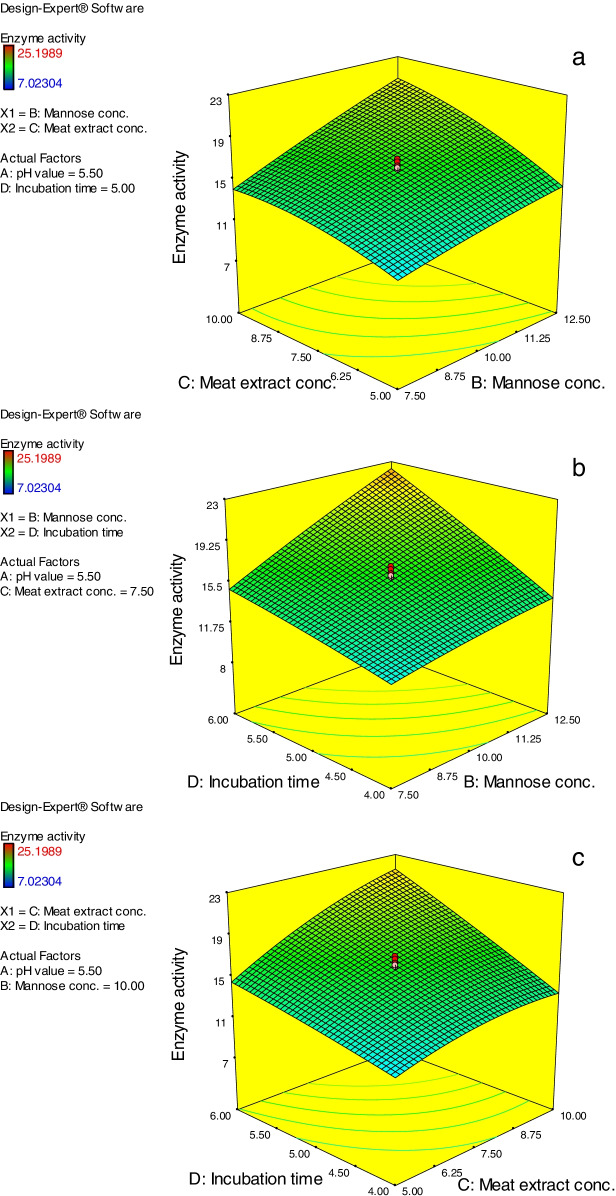
**(a**–**c)** Three-dimensional surface models of *A. niger* NRC114 α-galactosidase formation. For each figure, the absent two parameters were fixed at 0 levels (i.e. mannose conc., 10 g/L; meat extract, 7.5 g/L; and incubation time, 5 days) while the effect of the other two variables was measured

Results of three-dimensional (3D) surface models are cited in Figs. 5 and 6. The results obtained revealed the effectual role of the studied factors and their interaction effect on the *A. niger* NRC114 α-galactosidase production throughout utilizing enzyme activity as response value. The presented 3D graphs were created by fixing two of the four factors at their midpoints (pH value, 5.5; mannose concentration, 10 g/L; meat extract, 7.5 g/L; and incubation duration, 5 days) while measuring the influence of the other two parameters. These 3D surface models additionally verify the variations in caused effects between the considered factors on *A. niger* NRC114 α-galactosidase production, and their interconnect impact on the process.

Figure 5a shows the effect of varied pH values (*A*) and mannose concentrations (*B*) on the production of *A. niger* NRC114 α-galactosidase at a set meat extract concentration (7.5 g/L) (*C*) and incubation duration (5 days) (*D*). The steady rise in mannose concentration causes an increase in α-galactosidase production, but the increase in pH causes a decrease in α-galactosidase creation (Fig. 5a), which may be due to the effect of pH values on the 3D structure of the enzyme [21]. Figure 5b depicts the effect of different starting pH values (*A*) of the production medium and meat extract (*C*) concentrations on α-galactosidase synthesis by *A. niger* NRC114. The resulting response revealed that increasing the concentration of meat extract leads to an increase in α-galactosidase secretion, but increasing the pH values of the production medium has a detrimental influence on enzyme synthesis. Figure 5c depicts the effect of the interaction between starting pH values (*A*) and incubation duration (*D*). The resulting response curve shows that increasing the incubation duration (*D*) enhanced α-galactosidase production on a regular basis until 6 days, with pH 5.0–5.5 being the optimal pH value (Fig. 5c). In Fig. 6a, the impact of mannose (*B*) and meat extract (*C*) concentrations at fixed pH values of 5.5 (*A*) and an incubation duration of 5 days (*D*) was displayed. The curve depicts the direct influence of both factors on α-galactosidase formation, which rises as the concentrations of both parameters increase (Fig. 6a). Furthermore, the effect of mannose concentration (*B*) and incubation time (*D*) on *A. niger* NRC114 α-galactosidase production at the preset midpoints of pH value (5.5) and meat extract concentration of 7.5 g/L is shown in Fig. 6b, demonstrating the advantages of raising mannose concentration. Similarly, Fig. 6c depicts the results of correlations between meat extract concentration (*C*) and incubation duration (*D*) at fixed pH (5.5) and mannose level (10.0 g/L). The figure demonstrates that a minor increase in both factors (7.5 g/L and 5 days) caused an increase in *A. niger* NRC114 α-galactosidase formation.

In accordance with the obtained values from the designed model, the results of the optimization study applying the CCD design lead to the use of pH value of 4.73; mannose concentration of 12.5 g/L; meat extract concentration of 9.59 g/L; and incubation time of 6 days in order to get the desirability of enzyme activity units of 25.22 U/mL (4-fold more) compared to the 6.4 U/mL produced using a single factor per time study, which proves the models’ validity.

### Evaluation of improvement of soymilk properties (phenolic compounds and antioxidant activity) by α-galactosidase treatment

**Fig. 7:**
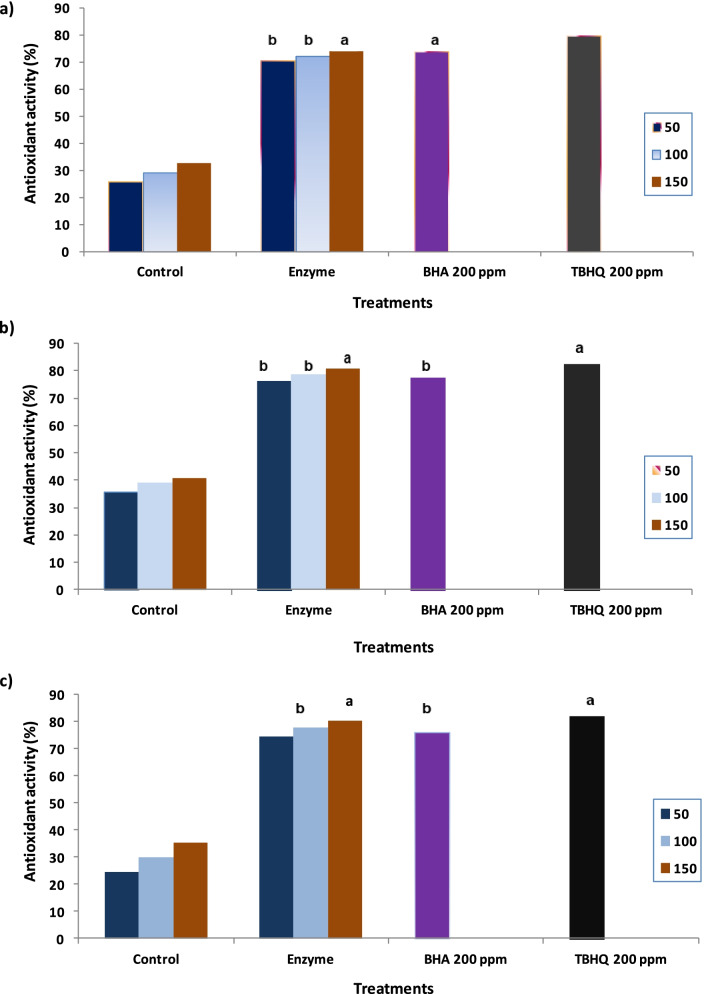
Antioxidant activity of raw and enzyme-treated soymilk at various concentrations as determined by (**a**) DPPH; (**b**) FARAP; and (**c**) β-carotene methods. The means of columns with the same letters are not significantly different (*P* ≤ 0.05)

Table 5 shows the efficiency of α-galactosidase on the treatment of soy milk by an increase in total phenols and flavonoids by 27.3% and 19.9%, respectively. Three methods have been used to evaluate the antioxidant activity of raw soy milk and that treated by α-galactosidase. The results in Fig. 7a show that DPPH free scavenging activity reflects the efficiency effect of enzyme addition to soymilk on increasing antioxidant activity compared to the control sample. The obtained results showed that the values for control and treated soymilk were 25.82 and 70.56%, respectively, compared to BHA and TBHQ, which had 73.9 and 79.8%, respectively (Fig. 7a). During the FRAP assay, the enzyme treatment of soymilk improved the antioxidant activity by 76.4% compared to the control sample (35.71%) and was non-significant in comparison with BHA and TBHQ at 100 μL and 150 μL, respectively (Fig. 7b). The ability of studied samples to neutralize the free radicals was measured using the β-carotene method and the data shown in Fig. 7c indicated the increase of the antioxidant activity with increasing the applied concentrations, which had a 24.5% in control soymilk and a significant increase (74.2%) was observed in soymilk after enzyme treatment.

**Table 5:** Phytochemicals, sugar composition, and sensory evaluation of soymilk samples before and after enzyme treatment

Parameter	Control soymilk	Treated soymilk
**Soymilk phytochemicals**		
Total phenols (mg/100 mL) GAE	152.87 ± 0.15^a^	194.63 ± 0.24
Total flavonoids (mg/100 mL) CA	62.87 ± 0.14	75.36 ± 0.18
**Sugar composition** (g/100 mL)		
Stachyose	1.63 ± 0.02	0.54 ± 0.01
Raffnose	0.86 ± 0.03	0.09 ± 0.01
Sucrose	3.49 ± 0.05	0.56 ± 0.01
Fructose	0.62 ± 0.01	4.16 ± 0.02
Glucose	n.d	3.12 ± 0.01
Galactose	1.37 ± 0.02	n.d
**Sensory evaluation**		
Appearance	6.58 ± 0.17	6.69 ± 0.13
Color	7.12 ± 0.08	7.25 ± 0.24
Flavor	3.25 ± 0.05	7.83 ± 0.12
Taste	3.41 ± 0.09	8.19 ± 0.31
Texture	4.12 ± 0.15	7.98 ± 0.16
Palatability	3.52 ± 0.12	8.24 ± 0.36

*GAE* gallic acid, *CA* catechin, *n.d* not detected

^a^Values are expressed as mean ± SD

### Hydrolysis of oligosaccharides in soy milk by α-galactosidase

**Fig. 8:**
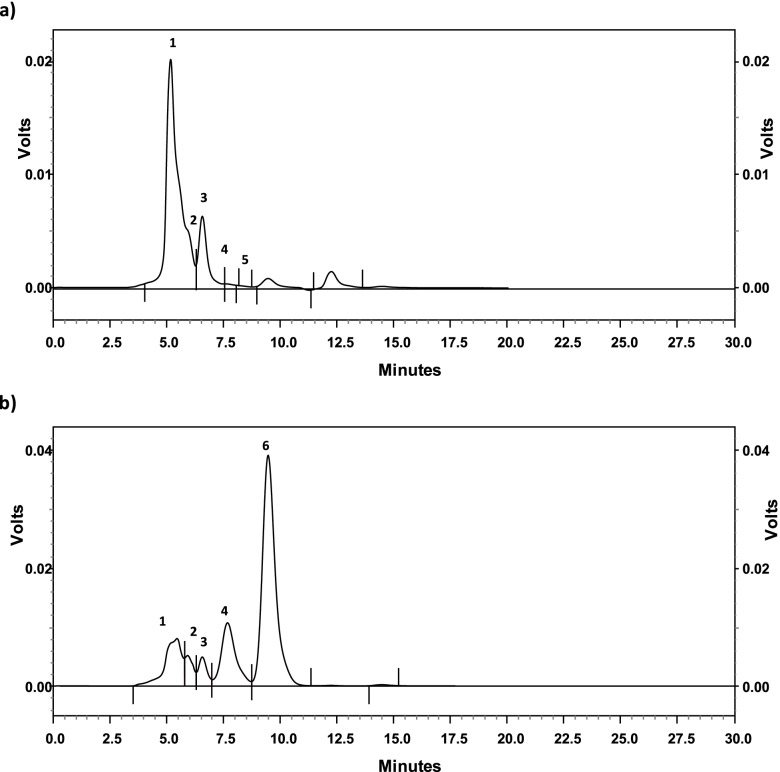
HPLC separation pattern of oligosaccharides in soymilk: (**a)** before treatment, and (**b)** after treatment with *A. niger* NRC114 α-galactosidase. The identified peaks were: 1-stachyose, 2-raffinose, 3-sucrose, 4-glucose, 5-galactose, and 6-fructose

The changes in different sugars of soymilk treated by α-galactosidase are given in Table 5 and Fig. 8. The data revealed that enzyme treatment resulted in a significant reduction of raffinose, stachyose, and sucrose. The most reduction was found in stachyose, sucrose, and raffinose after enzyme treatment compared to the control sample (Table 5). On the other hand, glucose and fructose showed a noticeable increase in the treated sample in comparison with the control sample.

### Evaluation of organoleptic quality in soymilk treated with α-galactosidase

The data of sensory evaluation revealed that soymilk treated with α-galactosidase was rated better than the control sample in terms of flavor, taste, and palatability (Table 5). The obtained data showed that enzyme treatment was effective in removing the beany flavor and taste in soymilk. In addition, the enzyme treatment has promoted a method for obtaining a sweet taste, a desirable flavor, and palatability. On the other hand, no significant variation was observed in terms of color and appearance between the control and enzyme-treated samples. The significant acceptability of the enzyme-treated sample in terms of taste, which had an 8.19 compared to a 3.41 in the control sample, may be due to the increase of sugars such as glucose and fructose after enzyme treatment (Table 5), while the raw sample contains significant concentrations of stachyose and raffinose.

### Effect of enzyme treatment on volatile compounds of soymilk

Forty-four volatile compounds in raw soymilk and the enzyme-treated samples were identified and detected with their available odor descriptions. They were grouped into seven chemical classes, including: (8) aldehydes, (8) alcohols, (6) esters, (6) acids, (7) ketones, (6) furans and furan derivatives, and (3) sulfur-containing compounds. Table 6 shows that the main volatile compounds in raw soymilk were acids (45.04%) which decreased to be 7.82% after the treatment by α-galactosidase. A remarkable decrease of acetic acid from 33.73% to 1.03% in the treated enzyme soymilk may explain the high scores of sensory evaluation mentioned in Table 5.

**Table 6:** Volatile compounds in soymilk before and after enzyme treatment

Volatile compound	KRI^a^	Soymilk ^b^		Description^c^
		Control	Enzyme treatment	
**Aldehydes**				
2-Methylbutanal	914	2.08	4.22	
3-Methylbutanal	918	0.45	1.12	Dark chocolate
Pentanal	975	0.59	1.33	Grass, green
Hexanal	1074	10.53	2.86	
Heptanal	1183	5.24	2.36	Rancid
Octanal	1282	4.35	2.43	Lemon, fruity
Nonanal	1387	5.87	4.87	Floral, citrus
Benzaldehyde	1513	5.14	0.84	Almond
**Subtotal**		**34.25**	**20.03**	
**Alcohols**				
Ethanol	931	0.48	4.69	
2-Butanol	1025	1.09	3.63	
2-Methyl-1-propanol	1088	2.53	1.35	
3-Pentanol	1102	0.13	1.13	
1-Butanol	1143	0.02	0.98	
1-Penten-3-ol	1157	0.21	2.96	
3-Methyl-1-butanol	1203	0.17	0.84	
1-Hexanol	1352	0.98	0.23	
**Subtotal**		**4.63**	**15.58**	
**Esters**				
Ethyl butanoate	1031	0.95	3.02	
Ethyl 2-methylbutanoate	1049	0.67	3.11	
Ethyl 3-methylbutanoate	1061	0.49	2.86	
Ethyl hexanoate	1224	1.05	1.21	
Ethyl lactate	1327	0.95	1.50	
Ethyl hexadecanoate	2205	0.41	1.03	
**Subtotal**		**4.52**	**12.73**	
**Acids**				
Acetic acid	1415	33.73	1.03	Sour
Propanoic acid	1513	4.51	1.94	
2-Methyl-propanoic acid	1538	1.48	0.89	
Butanoic acid	1597	3.38	1.27	
Pentanoic acid	1714	0.62	1.68	
Hexanoic acid	1822	1.32	1.01	
**Subtotal**		**45.04**	**7.82**	
**Ketones**				
2-Butanone	906	0.19	1.56	
2-Pentanone	973	2.14	0.90	
2-Hexanone	1072	0.50	1.34	
(E)-3-Penten-2-one	1116	0.14	1.73	
**Subtotal**		**2.97**	**5.53**	
**Furans and furan derivatives**				
2-Methylfuran	873	4.03	3.78	
2-Ethylfuran	954	1.15	0.7	Burnt
2-Butylfuran	1128	0.60	1.78	
2-Pentylfuran	1225	0.15	1.46	
Furfural	1457	0.86	11.60	
2-Furanmethanol	1654	0.35	7.02	
**Subtotal**		**7.14**	**26.34**	
**Sulfur-containing compounds**				
4-Methylthiazole	1625	0.19	0.25	
2-Acetylthiazole	1636	0.13	0.71	
3-(Methylthio)-1-propanol	1704	0.11	10.48	
**Subtotal**		**0.43**	**11.44**	

^a^Kovats retention index

^b^values are expressed as relative area percentages

^c^cited from Lv et al. [37]

Results in Table 6 clearly show the ability of the enzyme treatment to reduce the total aldehydes from 34.25 to 20.03%. The main identified aldehyde was hexanal, with a concentration of 10.53% and 2.86% in control and treated soymilk, respectively. The reduction in hexanal and hexanol after enzyme treatment correlated with the high score in sensory evaluation, especially in flavor and taste items (Table 5). The next major aldehydes in raw soymilk were nonanal and heptanal, which had 5.87% and 5.24%, respectively. This order changed to be nonanal and 2-methylbutanal, which had 4.87% and 4.22%, respectively, in enzyme-treated soymilk (Table 6). In addition, a dramatic decrease in benzaldehyde level of the treated soymilk was found to be 0.84% compared to the 5.14% in the untreated soymilk sample. Seven alcohols were identified and had a significant increase in enzyme treatment except for 2-methyl-1-propanol, which represented 2.53% compared to enzyme treatment (1.35%). The main alcohol that contributes to the beany-green odor in soy products was hexanol, which was reduced to 0.23% after enzyme treatment compared to 0.98% in the control soymilk.

After enzyme treatment, six furans and furan derivatives were identified with a pronounced concentration of 26.34% in comparison with the control sample, which represented 7.14% only (Table 6). The main furans in soymilk after enzyme treatment were furfural, 2-furanmethanol, and 2-methylfuran, which represented 11.60%, 7.02%, and 3.78%, respectively (Table 6). Three sulfur-containing compounds were identified in the current study, namely; 4-methylthiazole, 2-acetylthiazole, and 3-(methylthio)-1-propanol, which showed a remarkable increase after treatment of soymilk with enzyme, especially 3-(methylthio)-1-propanol, which recorded 10.48% in the enzyme sample compared to 0.11% in raw soymilk (Table 6).

## Discussion

*Aspergillus* species are found in house dust, humid environments, plant debris, and soil. *A. niger* characteristic conidiophores with abundant tubular phialides were recognized in the current study. Small subunit (18S) rRNA gene sequencing and hence phylogenetic tree of the isolate demonstrated the highest homology with other recorded *A. niger* strains in the GenBank (NCBI) database. The isolate was identified as *A. niger* NRC114 (Accession number: MW252165.1). The activity of α-galactosidase in *A. niger* NRC114 in the extra-cellular fraction exceeded by about 5.8-fold its presence in the endo-cellular one, whereas in our previous studies, the α-galactosidase enzyme was produced mainly endo-cellulary by different fungi [38, 39]. The high productivity of extra-cellular α-galactosidase increases the ability to use the enzyme in the treatment of soymilk to improve its properties.

Because of its direct impact on both fungal physiology and enzyme stability, the pH of the production medium is a key component that influences the secretion of fungal metabolites, particularly enzymes [21]. The optimum pH value (pH 5.5) of culture Dox’s medium to produce α-galactosidase by *A. niger* NRC114 is analogous to that produced by *Aspergillus terreus* [40] and varied with the reported enzyme of acidic pH value (pH 3.5) produced by *Bispora sp*. MEY-1 [41]. Mannose was superior, followed by maltose, and glucose to produce the enzyme, whereas galactose, lactose, and CMC were very poor carbon sources for assisting the formation of the enzyme, which may be explained as the fungus could not grow well on these compounds. Furthermore, the repression produced by CMC was attributable to an increase in viscosity, which reduces nutrition and oxygen availability, slows cell proliferation, and lowers metabolic rate [42]. On the other hand, α-galactosidase produced by *the Rhizopus oryzae* strain SUK exhibited the minimum level of production with mannose [18]. The nitrogen source is mainly an effective nutrient for enhancing enzyme production. Nitrogen sources have a significant impact on the physiology of microbes by influencing metabolic activities [21]. Here, various nitrogen sources, whether inorganic or organic (sodium nitrate, ammonium nitrate, ammonium tartarate, or meat extract), caused more or less the similar effect on the production of α-galactosidase, whereas yeast extract caused the lower productivity of the enzyme. Additionally, the addition of chickpea caused the highest formation of *A. niger* NRC114 α-galactosidase, followed by kidney bean powder and lupine powder. Dissimilarity was obvious between the present results and the reported effect of yeast extract that caused high production of α-galactosidase by the *Rhizopus oryzae* strain [18].

The response surface methodology was designed using the values of effective parameters (as obtained from one factor per time study) that influence the secretion of *A. niger* NRC114 α-galactosidase. ANOVA analysis revealed that the model (*R*^2^: 0.924) is significant; and pH value (*A*), mannose concentration (*B*), meat extract concentration (*C*), incubation time (*D*), *BC*, *BD*, *CD*, and C^2^ are significant model terms, and the model can be used to navigate the design space. The normal probability plot of the residuals is considered a noteworthy graphical way to explain the normality of data. The normality plot demonstrated that the relationship points are near to the diagonal line and the residuals are disseminated normally. This points to the fact that the predictable values of enzyme formation were well fitted with the investigational results [43]. The Box–Cox transformation is a way to develop uniformity of dispersion by promoting it to the power and adjusting it for statistical analysis. The power-transform is suited for creating a monotonic transformation of data via power functions [44]. In accordance with the obtained values from the designed model, the results of the optimization study applying the CCD design prove the models’ validity.

*A. niger* NRC114 α-galactosidase showed good efficiency in the treatment of soy milk by an increase in total phenols and flavonoids. Phenolics, protein, and soymilk oil can be considered the most important characteristics of soybeans and prepared products. Phenolic compounds are the secondary metabolites and most effective compounds that reduce the oxidative reactions in human and biological systems, which improves human health [45]. The efficiency of soy foods as potent antioxidants may be due to soy protein and protein-derived peptides, which increase after fermentation [46]. Another explanation for the improvement of antioxidant activity after enzyme treatment may be due to the formation of isoflavone aglycones during fermentation, which are more antioxidant compounds as mentioned by Marazza et al [47], who noted that genistein and daidzein were effective antiradical compounds after soymilk fermentation with the β-glucosidase enzyme. In addition, the data in the present study was confirmed by Abou-Dobara et al. [48], who found that Rayeb milk antioxidant activity increased after adding soymilk and explained the increase in antioxidant activity due to soybean protein. During the FRAP assay, the antioxidant compounds give up a single electron to the reagent of FRAP [49]. In a previous study carried out by Kim et al. [50], who referred to that soybean contains various phenolic compounds such as ferulic, vanillic, chlorogenic, and syringic acids. Therefore, the prepared soymilk from soybeans may contain significant concentrations of these phenolic compounds and be responsible for the antioxidant activity.

The obtained data in the current study referred to that treatment of soymilk by α-galactosidase may be a beneficial and practical approach to overcome the flatulence due to the presence of oligosaccharides. In accordance with our results, Kapnoor and Mulimani [51] reported that α-galactosidase from *A. oryzae* may be applicable in degrading stacchyose and raffinose present in soy milk*.* Also, α-galactosidase purified from *Coriolus versicolor* showed an ability to hydrolyze raffinose oligosaccharides completely to yield galactose and sucrose [52] and α-galactosidase from *Tremella aurantia alba* [53]. Organoleptic evaluation in any experimental investigation plays a domestic role to determine consumer acceptability. Soymilk treated with α-galactosidase was rated better than control in terms of flavor, taste, and palatability. The main contributor to the green-beany aroma in soymilk comes from hexanal. Therefore, several attempts were carried out to eliminate or reduce the hexanal content in soybeans and soy products [54, 55]. The reduction in hexanal and hexanol after enzyme treatment correlated with the high score in sensory evaluation, especially in flavor and taste items. The obtained results are in accordance with Chua et al. [56] who found that aldehydes, particularly hexanal and alcohols, 1-hexanol, were the main volatile compounds in soy (tofu) whey. After enzyme treatment, six furans and furan derivatives were identified. These volatile compounds are derived from lipid oxidation during the Maillard reaction and are responsible for caramel-like, fruity, and sweet notes in foods [57]. The high concentration of these furans may cover the effect of 2-pentylfuran, which has a beany or grassy aroma [58].

Our findings have a high level of importance for future studies. In the current study, the crude enzyme production was improved, demonstrating that competent treatment of soymilk with α-galactosidase may be a helpful and practical way to overcome flatulence caused by the presence of oligosaccharides. So, in the future, enzyme purification and characterization are very significant and should be addressed in future studies to examine its catalytic capabilities. These catalytic applications may be expanded to many biomedical aspects such as blood group conversion, Fabry disease therapy, and elimination of α-gal type immunogenic epitopes in xeno-transplantation.

## Conclusion

*A. niger* NRC114, an isolated local fungus, was found to be capable of secreting a toxin-free extracellular α-galactosidase. Extracellular enzyme formation is considered an advantage due to its mass production compared to endocellular one. Optimization production of the enzyme by the traditional method using a single factor at a time and also the CCD model were achieved and succeeded in enhancing the enzyme production by about 4-fold. The produced enzyme proved its potency for the improvement of the undesirable characteristics of raw soymilk. The current study’s findings indicate that treating soymilk with α-galactosidase may be a beneficial and practical approach to overcoming flatulence caused by the presence of oligosaccharides in raw soymilk. Besides, reduction of the main volatile compounds in soymilk after enzyme treatment was correlated with a high score in sensory evaluation, especially in flavor and taste items.

## Data Availability

All data generated or analyzed during this study are included in this published article.
